# Characterization of alphasatellites associated with monopartite begomovirus/betasatellite complexes in Yunnan, China

**DOI:** 10.1186/1743-422X-7-178

**Published:** 2010-08-03

**Authors:** Yan Xie, Peijun Wu, Pei Liu, Huanran Gong, Xueping Zhou

**Affiliations:** 1 State Key Laboratory of Rice Biology, Institute of Biotechnology, Zhejiang University, Hangzhou 310029, P.R. China

## Abstract

**Background:**

Alphasatellites are single-stranded molecules that are associated with monopartite begomovirus/betasatellite complexes.

**Results:**

Alphasatellites were identified in begomovirus-infected plant samples in Yunnan, China. All samples that contained alphasatellites also contained betasatellites, but only some samples that contained betasatellites contained alphasatellites. Thirty-three alphasatellites were sequenced, and they ranged from 1360 to 1376 nucleotides. All alphasatellites contain 3 conserved features: a single open reading frame (Rep), a conserved hairpin structure, and an adenine-rich (A-rich) region. On the basis of the phylogenetic tree of the complete nucleotide sequences, the alphasatellites were divided into 3 types with one exception. Type 1 was associated with *Tomato yellow leaf curl China virus *(TYLCCNV)/*Tomato yellow leaf curl China betasatellite *(TYLCCNB) complex. Type 2 was associated with *Tobacco curly shoot virus *(TbCSV)/*Tobacco curly shoot betasatellite *(TbCSB) complex. Type 3 was associated with TbCSV/*Ageratum yellow vein betasatellite *(AYVB) complex. Within each type, nucleotide sequence identity ranged from 83.4 to 99.7%, while 63.4-81.3% identity was found between types. Mixed infections of alphasatellites associated with begomovirus/betasatellite complexes were documented.

**Conclusions:**

Our results validate that alphasatellites are only associated with begomovirus/betasatellite complexes. Thirty-three sequenced alphasatellites isolated from Yunnan Province, China were divided into 3 types--each associated with a specific begomovirus/betasatellite complex. Mix-infections of alphasatellite molecules may not be unusual.

## Background

Geminiviruses are a group of plant viruses characterized by their geminate shape and the size of their particles, which encapsidate a circular single-stranded DNA genome. Due to their wide host range and high frequency of genome variation, geminiviruses cause substantial yield losses in many crops, including tomato, cassava, and cotton, throughout tropical and sub-tropical regions worldwide [1,2]. The majority of geminiviruses described belong to the genus *Begomovirus *in the family *Geminiviridae*, they are transmitted by the whitefly, *Bemisia tabaci *[3]. Most begomoviruses have 2 components, which are referred to as DNA-A and DNA-B, both are essential for virus proliferation. Many species only have a single genomic component that resembles DNA-A [1,3]. Some monopartite begomoviruses are associated with betasatellites (formerly DNAβ), which affect the replication of their respective helper begomoviruses and alter the symptoms induced in some host plants [4-9]. Analysis of betasatellites reveals that they are approximately half the size of the genomic DNA, and except for a conserved hairpin structure and a TAATATTAC loop sequence, they have little sequence similarity to either the DNA-A or DNA-B molecules of begomoviruses. Betasatellites require begomoviruses for replication, encapsidation, insect transmission, and movement in plants [10].

Alphasatellites (formerly DNA1) are circular, single-stranded DNA molecules associated with begomovirus/betasatellite complexes [11-15]. Alphasatellites are approximately half the size of begomovirus DNA and encode a rolling-circle replication initiator protein similar to nanoviruses. Consequently, alphasatellites are capable of self-replication in host plants, but require helper begomoviruses for movement in plants as well as insect transmission.

In China, several begomoviruses are reported to infect squash, tobacco, ageratum, tomato, and malvastrum; many begomovirus isolates are associated with betasatellites, and co-evolution of betasatellites with their helper viruses has been documented [9,16-21]. In this report, we identify 33 alphasatellites from Yunnan Province, China, and demonstrate that they can be classified into 3 types--each associated with a specific begomovirus/betasatellite complex.

## Results

### Alphasatellites associated with various begomovirus/betasatellite complexes in Yunnan, China

More than 300 plant samples exhibiting begomovirus-like symptoms, including *Ageratum conyzoides*, *Malvastrum coromandelianum*, and tobacco, tomato, and squash plants, were collected from widely separated locations in Yunnan. The majority of these isolates were found to be infected with 1 or 2 of the following 7 viruses: *Tobacco curly shoot virus *(TbCSV) [7], *Tobacco leaf curl Yunnan virus *(TbLCYNV) [19], *Tomato yellow leaf curl China virus *(TYLCCNV) [5], *Tomato yellow leaf curl Thailand virus *(TYLCTHV) [16], *Malvastrum yellow vein virus *(MYVV) [17], *Malvastrum yellow vein Yunnan virus *(MYVYNV) [22], and *Squash leaf curl Yunnan virus *(SLCYNV) [20]. Some of these viruses are known to be associated with betasatellites (Table 1). Alphasatellites were identified from tobacco, tomato, ageratum, and malvastrum plants infected by TbCSV, TYLCCNV, TbCSV + TYLCCNV, TbCSV + TbLCYNV, TYLCCNV + TYLCTHV, TbCSV + MYVV, and TbCSV + MYVYNV. However, alphasatellites were not found in tomato plants infected by TYLCTHV, tobacco plants infected by TbLCYNV, malvastrum plants infected by MYVV or MYVYNV, or squash plants infected by SLCYNV (Table 1). When tested by PCR, all samples that had alphasatellites were found to be associated with betasatellites, however, only some samples that had betasatellites were found to be associated with alphasatellites. A high proportion of samples infected by TbCSV/*Tobacco curly shoot betasatellite *(TbCSB) complex (50%) and TYLCCNV/*Tomato yellow leaf curl China betasatellite *(TYLCCNB) complex (42.9%) were associated with alphasatellites, whereas no samples infected by TYLCTHV/*Tomato yellow leaf curl Thailand betasatellite *(TYLCTHB), MYVV/*Malvastrum yellow vein betasatellite *(MYVB), or MYVYNV/*Malvastrum yellow vein Yunnan betasatellite *(MYVYNB) complexes contained alphasatellites (Table 1). TbLCYNV and SLCYNV isolates were not associated with betasatellites; additionally, alphasatellites were not detected in samples infected by TbLCYNV or SLCYNV (Table 1). Furthermore, we found that the severity of symptoms appearing in plants was similar whether or not they were infected with alphasatellites.

**Table 1:** Association of begomovirus with alphasatellite and betasatellite

Begomovirus	No. of total isolates	No. of isolates having betasatellite	No. of isolates having alphasatellite	No. of isolates having alphasatellite and betasatellite
TbCSV	36	14	7	7
TYLCCNV	56	56	24	24
TYLCTHV	5	5	0	0
TbLCYNV	18	0	0	0
MYVV	16	16	0	0
MYVYNV	8	2	0	0
SLCYNV	1	0	0	0
TbCSV+TYLCCNV	6	6	5	5
TbCSV+TbLCYNV	6	6	6	6
TYLCCNV+TYLCTHV	4	3	3	3
TbCSV+MYVV	3	3	3	3
TbCSV+MYVYNV	2	2	2	2

### Sequence analysis of alphasatellites

The complete nucleotide sequences of the 23 alphasatellites from tobacco, 3 from tomato, 2 from ageratum, and 5 from malvastrum plants (total: 33) were determined to be 1360 to 1376 nucleotides (nts) in length--this is longer than betasatellites, which range from 1333 to 1355 nts in length. The sequences of these 33 alphasatellites have been submitted to GenBank under the accession numbers AJ579345-AJ579361, AJ888445-AJ888455, and FN678899-FN678903 (Table 2). The alphasatellites are named according to their sample number; thus, Y35A refers to alphasatellites from sample Y35.

**Table 2:** Origin and features of alphasatellite molecules

Clone	Plant species	Origin (town/year)	Helper begomovirus	Associated betasatellite	Size	Accession number
Y89A	Tobacco	Baoshan/2002	TYLCCNV	TYLCCNB	1360	AJ579358
Y8A	Tobacco	Honghe/1999.08	TYLCCNV	TYLCCNB	1363,1367	AJ579353; AJ888446
Y36A	Tobacco	Honghe/2001.06	TYLCCNV	TYLCCNB	1363	AJ579354
Y38A	Tobacco	Honghe/2001.06	TYLCCNV	TYLCCNB	1361	AJ579355
Y39A	Tobacco	Honghe/2001.06	TYLCCNV	TYLCCNB	1365	AJ579356
Y261A	Tobacco	Baoshan/2004.08	TYLCCNV	TYLCCNB	1363	AJ888448
Y244A	Tobacco	Honghe/2004.08	TYLCCNV	TYLCCNB	1361	AJ888449
Y248A	Tobacco	Honghe/2004.08	TYLCCNV	TYLCCNB	1362	AJ888450
Y240A	Tobacco	Wenshan/2004.08	TYLCCNV	TYLCCNB	1364	AJ888451
Y241A	Tobacco	Wenshan/2004.08	TYLCCNV	TYLCCNB	1362	AJ888452
Y70A	Tomato	Baoshan/2002.01	TYLCCNV TYLCTHV	TYLCCNB TYLCTHB	1363	AJ579359
Y71A	Tomato	Baoshan/2002.01	TYLCCNV TYLCTHV	TYLCTHB	1365	AJ888447
Y72A	Tomato	Baoshan/2002.01	TYLCCNV TYLCTHV	TYLCTHB	1364	AJ579360
Y35A	Tobacco	Baoshan/2001.04	TbCSV	TbCSB	1367	AJ579345
Y99A	Tobacco	Baoshan/2002.01	TbCSV	TbCSB	1371	AJ579347
Y130A	Tobacco	Baoshan/2002.01	TbCSV	TbCSB	1369	AJ579348
Y135A	Tobacco	Baoshan/2002.01	TbCSV	TbCSB	1367	AJ579350
Y283A	Malvastrum	Baoshan/2004.08	TbCSV	TbCSB	1370	FN678903
Y143A	Tobacco	Baoshan/2002.01	TbCSV TbLCYNV	TbCSB	1370	AJ579361
Y290A	Tobacco	Baoshan/2004.08	TbCSV TbLCYNV	TbCSB	1371	AJ888453
Y115A	Tobacco	Baoshan/2002.01	TbCSV TYLCCNV	TbCSB	1368	AJ579346
Y87A	Tobacco	Baoshan/2002.01	TbCSV TYLCCNV	TYLCCNB	1367, 1361	AJ579357; AJ888445
Y146A	Tobacco	Baoshan/2002.01	TbCSV TYLCCNV	TYLCCNB	1370	AJ579352
Y132A	Tobacco	Baoshan/2002.01	TbCSV TbLCYNV	AYVB	1375	AJ579349
Y273A	Ageratum	Baoshan/2004.08	TbCSV TbLCYNV	AYVB	1375	AJ888454
Y276A	Ageratum	Baoshan/2004.08	TbCSV TbLCYNV	AYVB	1375	AJ888455
Y137A	Tobacco	Baoshan/2002.01	TbCSV TbLCYNV	AYVB TbCSB	1373	AJ579351
Y277A	Malvastrum	Baoshan/2004.08	TbCSV MYVYNV	AYVB MYVYNB	1374	FN678899
Y278A	Malvastrum	Baoshan/2004.08	TbCSV MYVYNV	AYVB MYVYNB	1374	FN678900
Y216A	Malvastrum	Yuxi/2003.11	TbCSV MYVV	MYVB	1376	FN678901
Y249A	Malvastrum	Honghe/2004.08	TbCSV MYVV	MYVB	1374	FN678902

Nucleotide sequence comparisons show that the 33 alphasatellites can be divided into 3 types (Table 3). Type 1 consists of 9 samples infected by TYLCCNV and 3 samples infected by TYLCCNV + TYLCTHV; overall nucleotide sequence identity is 83.4-99.7%. Type 2 consists of 5 samples infected by TbCSV, 2 samples infected by TbCSV + TbLCYNV, and 3 samples infected by TbCSV + TYLCCNV; the sequences in type 2 share 91.4-98.2% identity. Type 3 consists of samples mix-infected by TbCSV and other begomoviruses, including 4 samples inflected by TbCSV + TbLCYNV, 2 by TbCSV + MYVYNV, and 2 by TbCSV + MYVV; sequences in type 3 share 90.3-99.6% identity. The overall nucleotide sequence identity between types 1 and 2 is 75.9-81.3%, 63.4-72.0% between types 1 and 3 and 69.3-75.5% between types 2 and 3. Y89A is distinct among the 33 alphasatellites and shares only 69.3-79.5% nucleotide sequence identity with alphasatellites of the 3 types. A relatively lower sequence identity (58.9-71.8%) exists between the present 33 and previously reported alphasatellites (data not shown).

**Table 3:** Percentage nucleotide sequence identity (top right) and predicted amino acid sequence similarities Rep (bottom left) of alphasatellite components

	1*	2	3	4	5	6	7	8	9	10	11	12	13	14	15	16	17	18	19	20	21	22	23	24	25	26	27	28	29	30	31	32	33
Y72A		**99.3**	**99.1**	**98.5**	**97.5**	**90.2**	**90.7**	**91.0**	**90.5**	**88.6**	**84.8**	**85.8**	**83.9**	**84.1**	79.5	80.4	79.0	79.0	77.7	80.5	80.0	80.2	80.3	80.1	**74.6**	68.4	68.3	70.3	67.7	70.6	70.5	66.7	66.6
Y70A	**98.7**		**99.7**	**99.1**	**98.1**	**90.5**	**90.6**	**90.9**	**90.8**	**88.9**	**85.1**	**86.1**	**84.1**	**84.7**	79.8	80.6	79.5	76.5	80.9	80.8	80.5	80.6	80.6	80.5	**78.0**	68.7	68.5	68.5	68.0	68.8	68.7	.66.8	67.1
Y8A-5	**99.0**	**99.4**		**99.0**	**97.9**	**90.4**	**90.4**	**90.7**	**90.6**	**88.8**	**85.0**	**86.0**	**83.9**	**84.6**	79.6	80.5	79.4	76.4	80.7	80.6	80.3	80.5	80.5	80.4	**77.8**	68.7	68.5	68.3	68.0	68.7	68.5	66.8	67.2
Y71A	**99.4**	**99.7**	**99.7**		**97.4**	**90.1**	**90.0**	**90.3**	**90.0**	**88.2**	**84.3**	**85.3**	**83.5**	**84.2**	78.5	80.0	78.5	78.7	79.7	79.7	79.6	77.4	79.9	79.7	**75.4**	69.7	69.6	69.9	68.3	68.6	67.8	67.3	66.9
Y87A-7	**97.2**	**97.5**	**97.8**	**98.1**		**90.6**	**91.2**	**91.6**	**90.6**	**88.6**	**84.6**	**85.7**	**83.9**	**84.2**	79.5	80.3	79.4	79.8	79.7	80.5	77.5	80.3	80.7	77.8	**78.4**	70.1	70.1	70.2	67.7	70.0	69.9	67.3	67.3
Y261A	**96.8**	**97.1**	**97.1**	**97.5**	**96.8**		**90.4**	**90.6**	**89.7**	**88.4**	**84.6**	**85.4**	**84.4**	**83.8**	80.0	80.8	76.9	79.5	79.7	80.7	80.9	80.5	79.8	80.6	**78.1**	70.4	70.4	70.1	66.5	67.3	67.1	68.4	66.1
Y244A	**97.1**	**97.5**	**97.5**	**97.8**	**97.1**	**98.1**		**99.3**	**93.6**	**91.9**	**87.0**	**87.7**	**86.3**	**85.7**	80.1	80.7	80.0	80.2	80.3	80.5	80.5	80.2	80.6	81.0	**79.5**	68.8	68.7	70.2	68.2	68.6	68.6	69.9	67.2
Y248A	**97.1**	**97.5**	**97.5**	**97.8**	**97.1**	**98.1**	**100**		**93.2**	**91.7**	**87.0**	**87.7**	**86.3**	**85.7**	80.0	80.6	79.9	80.1	76.9	80.2	79.8	80.1	79.7	80.1	**79.2**	70.7	70.5	70.9	69.2	70.8	68.6	69.8	64.6
Y36A	**96.2**	**96.5**	**96.8**	**97.1**	**96.2**	**96.8**	**98.1**	**98.1**		**96.3**	**88.9**	**90.9**	**85.9**	**85.2**	80.6	81.1	80.4	80.2	80.7	80.9	80.1	80.6	81.3	81.0	**75.1**	67.4	67.4	70.8	67.4	71.2	71.0	69.8	66.3
Y38A	**95.6**	**95.9**	**95.9**	**96.2**	**95.6**	**96.8**	**98.1**	**98.1**	**98.1**		**89.4**	**91.8**	**84.3**	**83.4**	80.1	80.4	81.3	79.9	79.9	80.0	79.9	80.2	77.4	77.6	**74.9**	68.8	70.8	70.1	68.3	67.2	68.4	67.7	63.4
Y8A-6	**93.7**	**94.0**	**94.0**	**94.3**	**93.3**	**94.6**	**95.9**	**95.9**	**94.6**	**95.2**		**96.0**	**89.5**	**89.1**	77.3	78.9	78.7	77.5	77.7	77.8	77.3	78.2	77.7	77.8	**74.1**	68.2	67.7	67.4	67.1	67.9	67.6	66.4	65.3
Y39A	**93.7**	**94.0**	**94.0**	**94.3**	**93.3**	**94.6**	**95.9**	**95.9**	**95.2**	**95.2**	**98.7**		**90.2**	**89.4**	79.0	77.5	79.9	79.6	79.9	79.3	79.0	77.1	79.5	79.9	**74.9**	70.9	70.4	70.1	68.3	65.3	65.1	68.0	66.9
Y240A	**93.7**	**94.0**	**94.0**	**94.3**	**93.3**	**93.3**	**94.6**	**94.6**	**94.3**	**93.3**	**97.5**	**97.5**		**97.8**	76.1	80.0	77.9	78.5	75.9	75.9	77.9	79.2	78.0	78.2	**76.2**	71.9	69.4	69.3	71.3	69.3	69.4	70.5	70.7
Y241A	**93.7**	**94.0**	**94.0**	**94.3**	**93.0**	**93.0**	**94.3**	**94.3**	**94.0**	**93.0**	**97.1**	**97.1**	**99.7**		79.1	80.2	78.3	78.6	78.7	79.1	78.1	79.4	78.6	78.1	**76.4**	72.0	71.5	70.6	71.3	71.5	71.7	71.0	67.8
Y283A	93.0	93.3	93.3	93.7	93.3	92.4	93.7	93.7	93.7	92.7	90.5	91.1	91.7	91.7		**97.6**	**95.5**	**96.1**	**94.6**	**96.9**	**96.0**	**95.6**	**94.0**	**94.4**	**71.5**	75.5	75.3	73.4	73.0	74.1	74.0	71.5	70.6
Y290A	93.7	94.0	94.0	94.3	93.7	93.0	94.3	94.3	94.3	93.3	91.7	92.4	93.0	93.0	**98.1**		**95.1**	**95.8**	**94.4**	**96.4**	**95.3**	**95.1**	**93.6**	**93.5**	**73.5**	75.3	75.1	73.1	72.0	73.2	73.1	71.4	70.4
Y115A	93.3	93.7	93.7	94.0	93.7	92.7	94.0	94.0	94.0	94.3	91.4	91.4	92.1	92.1	**97.8**	**97.8**		**98.1**	**95.2**	**96.1**	**95.4**	**93.2**	**92.4**	**93.3**	**69.8**	73.1	72.9	72.6	72.4	73.3	72.4	70.2	69.7
Y130A	93.0	93.3	93.3	93.7	93.3	92.4	93.7	93.7	93.7	92.7	90.5	91.1	91.7	91.7	**97.5**	**97.5**	**98.4**		**96.4**	**96.8**	**96.5**	**93.9**	**93.3**	**94.3**	**71.7**	72.9	72.8	72.2	72.4	72.0	72.0	69.3	69.7
Y143A	92.7	93.0	93.0	93.3	93.0	92.1	93.3	93.3	93.3	92.4	90.2	90.8	91.4	91.4	**96.5**	**96.5**	**97.5**	**97.5**		**95.3**	**94.8**	**93.4**	**93.7**	**93.7**	**70.7**	73.3	72.8	73.4	72.8	73.4	73.0	69.9	70.0
Y146A	94.0	94.3	94.3	94.6	94.0	93.3	94.6	94.6	94.6	93.7	91.7	92.4	93.0	93.0	**97.8**	**98.4**	**98.1**	**97.8**	**96.8**		**97.1**	**94.9**	**95.2**	**95.3**	**72.7**	73.9	73.5	72.7	72.0	72.6	72.5	70.4	69.9
Y135A	93.7	94.0	94.0	94.3	93.7	93.3	94.3	94.3	94.3	93.3	91.4	92.1	92.7	92.7	**97.5**	**98.1**	**97.8**	**97.5**	**96.5**	**99.7**		**93.5**	**95.2**	**95.8**	**72.0**	73.7	72.9	72.9	72.4	72.2	72.1	69.7	70.2
Y99A	94.0	94.3	94.3	94.6	94.0	93.3	94.6	94.6	94.6	93.7	91.4	92.1	92.7	92.7	**98.4**	**98.4**	**97.8**	**97.5**	**96.5**	**98.1**	**97.8**		**91.4**	**91.4**	**70.8**	74.8	74.6	73.8	72.1	73.1	73.0	71.0	69.7
Y35A	92.4	92.7	92.7	93.0	92.4	91.7	93.0	93.0	93.3	92.4	90.2	90.8	91.4	91.4	**95.9**	**96.5**	**96.2**	**95.9**	**94.9**	**97.8**	**97.5**	**96.2**		**98.2**	**71.5**	75.1	74.7	74.2	73.7	74.0	74.0	71.8	71.5
Y87A-2	92.7	93.0	93.0	93.3	92.7	92.1	93.3	93.3	93.7	92.7	90.5	91.1	91.7	91.7	**96.5**	**97.1**	**96.8**	**96.5**	**96.2**	**98.4**	**98.1**	**96.8**	**98.7**		**71.3**	75.0	74.5	74.0	73.7	73.8	73.7	71.5	72.3
Y89A	**89.5**	**89.8**	**89.8**	**90.2**	**90.2**	**89.8**	**90.8**	**90.8**	**90.8**	**89.8**	**91.1**	**91.1**	**93.0**	**92.7**	**90.2**	**90.8**	**89.8**	**89.5**	**88.6**	**90.8**	**90.5**	**90.8**	**90.8**	**90.5**		**70.2**	**70.1**	**69.9**	**69.4**	**70.7**	**70.4**	**69.6**	**69.3**
Y132A	89.2	89.6	89.8	90.2	89.2	88.6	90.2	90.2	89.9	89.2	90.2	90.8	90.8	90.8	91.4	92.4	90.8	90.8	90.2	91.4	91.1	91.7	90.8	91.1	**89.5**		**99.5**	**97.5**	**97.8**	**98.4**	**98.3**	**92.1**	**91.5**
Y273A	89.5	89.8	89.8	90.2	89.5	88.6	90.2	90.2	90.2	89.2	90.2	90.8	90.8	90.8	91.4	92.4	90.8	90.8	90.2	91.4	91.1	91.7	90.8	91.1	**89.5**	**100**.		**97.3**	**97.7**	**98.2**	**98.0**	**91.6**	**91.2**
Y276A	89.2	89.5	89.5	89.8	89.5	88.3	89.8	89.8	89.8	88.9	89.8	90.5	90.5	90.5	91.4	92.4	90.8	90.8	90.2	91.4	91.1	91.7	90.8	91.1	**89.2**	**99.4**	**99.4**		**98.3**	**98.9**	**98.8**	**91.2**	**91.3**
Y137A	89.2	89.6	89.8	90.2	89.2	88.6	90.2	90.2	89.9	89.2	90.2	90.8	90.8	90.8	91.1	92.4	90.8	90.8	90.2	91.4	91.1	91.4	90.8	91.1	**89.5**	**99.4**	**99.7**	**99.0**		**99.3**	**99.0**	**91.3**	**91.3**
Y277A	89.5	89.8	89.8	90.2	89.5	88.6	90.2	90.2	90.2	89.2	90.2	90.8	90.8	90.8	91.4	92.4	90.8	90.8	90.2	91.4	91.1	91.7	90.8	91.1	**89.5**	**100**	**100**	**99.4**	**99.7**		**99.6**	**91.7**	**91.3**
Y278A	89.5	89.8	89.8	90.2	89.5	88.6	90.2	90.2	90.2	89.2	90.2	90.8	90.8	90.8	91.4	92.4	90.8	90.8	90.2	91.4	91.1	91.7	90.8	91.1	**89.5**	**100**	**100**	**99.4**	**99.7**	**100**		**91.9**	**91.6**
Y216A	88.6	88.9	88.9	89.2	88.3	87.3	88.9	88.9	88.9	87.9	89.5	90.2	90.8	90.8	90.8	91.7	90.2	90.2	89.5	90.8	90.5	91.1	90.2	90.5	**89.5**	**98.1**	**98.1**	**97.8**	**97.8**	**98.1**	**98.1**		**90.3**
Y249A	87.3	87.6	87.6	87.9	87.3	86.3	87.9	87.9	87.9	87.0	87.9	88.6	89.2	89.2	89.5	90.5	88.9	88.9	88.3	89.5	89.2	89.8	88.9	89.2	**88.6**	**96.8**	**96.8**	**96.5**	**96.5**	**96.8**	**96.8**	**97.1**	

*1: Y72A; 2: Y70A; 3: Y8A-5; 4: Y71A; 5: Y87A-7; 6: Y261A; 7: Y244A; 8: Y248A; 9: Y36A; 10: Y38A; 11: Y8A-6; 12: Y39A; 13: Y240A; 14: Y241A; 15: Y283A; 16: Y290A; 17: Y115A; 18: Y130A; 19: Y143A; 20: Y146A; 21: Y135A; 22: Y99A; 23: Y35A; 24: Y87A-2; 25: Y89A; 26: Y132A; 27: Y273A; 28: Y276A; 29: Y137A; 30: Y277A; 31: Y278A; 32: Y216A; 33: Y249A.

**Figure 1:**
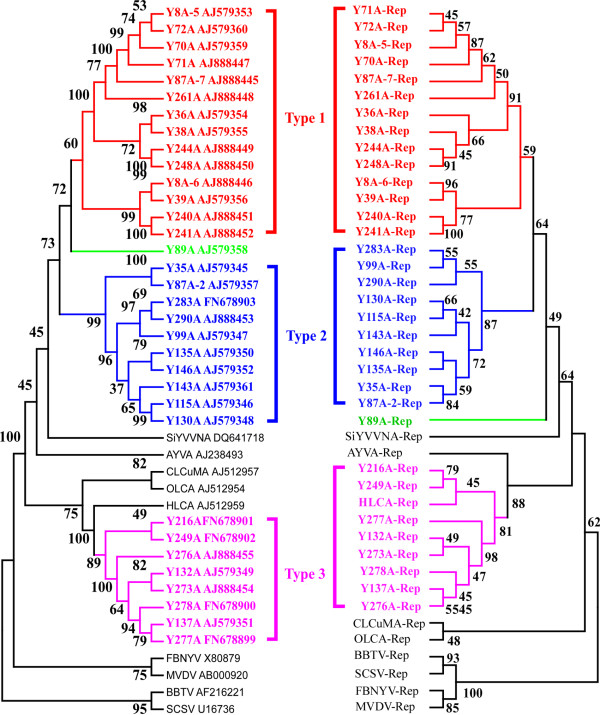
**Phylogenetic trees based on alignments of the complete nucleotide sequences (left) or Rep amino acid sequences (right) of alphasatellite components**. Trees were generated using the Neighbor-joining method using MEGA 4. Horizontal distances are proportional to sequence distances and vertical distances are arbitrary. The numbers at each branch indicate the percentage of 1000 bootstrap, which supports the grouping at each node.

Further analysis revealed that type 1 alphasatellites can be further classified into 3 separate subtypes. One subtype contains 5 alphasatellites (Y70A, Y71A, Y72A, Y87A-7, and Y261A) from Baoshan District and Y8A-5 from Honghe District. The second subtype consists of 4 alphasatellites (Y36A, Y38A, Y244A, and Y248A) from Honghe District. The third branch consists of 4 alphasatellites, among them, Y8A-6 and Y39A were from Honghe District, and Y240A and Y241A were from Wenshan District. There are 2 subtypes of type 2: one consists of 8 alphasatellites (Y99A, Y115A, Y130A, Y135A, Y143A, Y146A, Y283A, and Y290A) and the other consists of 2 alphasatellites (Y35A and Y87A-2); all isolates were from Baoshan District. Type 3 molecules consist of 8 alphasatellites (Y132A, Y137A, Y216A, Y249A, Y273A, Y276A, Y277A and Y278A) from Baoshan, Honghe, and Yuxi districts, and cluster with *Hibiscus leaf curl virus *(HLCA) (Figure 1, left). The relationship dendrogram of alphasatellites and nanoviruses reveals that alphasatellites form a large branch, while nanovirus DNA sequences form separate branches (Figure 1, left).

### Structural features of alphasatellites

**Figure 2:**
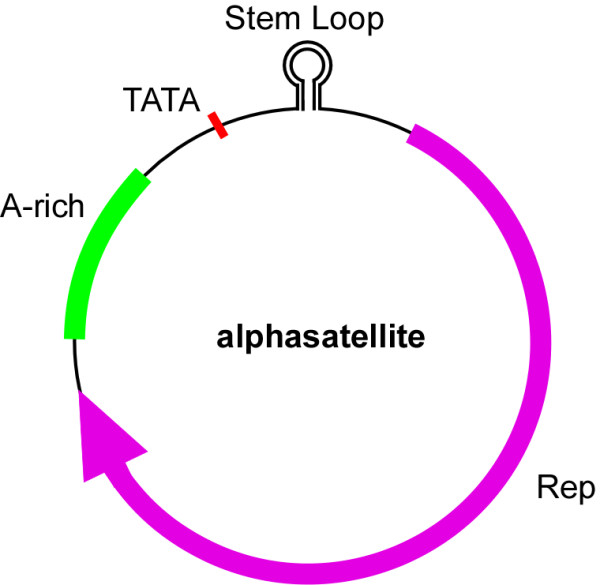
**Genomic structure of alphasatellite components**.

**Figure 3:**
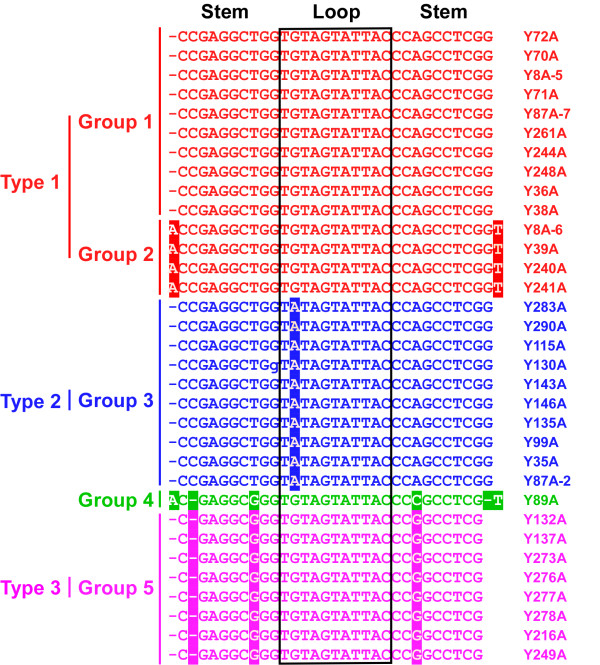
**Alignment of the hairpin sequences of alphasatellite components**. Positions of the stem and loop sequences are indicated. Spaces (-) are introduced to optimize the alignment.

All 33 alphasatellites contain 3 conserved features: a conserved hairpin structure, a single open reading frame, and an adenine-rich (A-rich) region (Figure 2). The highly conserved structure contains a predicted hairpin structure with a loop that includes the nonanucleotide, TAGTATTAC, which is common to nanoviruses and is similar to the TAATATTAC sequence of geminiviruses. For both geminiviruses and nanoviruses, this sequence contains the origin of replication, and is nicked by Rep to initiate virion-strand DNA replication. Alignment analysis indicates that alphasatellite hairpin structures fall into 5 groups. Groups 1 and 2 contain 10 and 4 alphasatellites, respectively; all alphasatellites in groups 1 and 2 belong to type 1 and share the same loop sequences, but in different stems. Group 3 has 10 alphasatellites which belong to type 2. Alphasatellites in groups 1 and 3 share the same stem sequences excluding one different (G/A) nucleotide in the loop. Group 4 contains only 1 alphasatellite (Y89A), which is distinct from the other 32 owing to its unique stem sequence. Group 5 contains 8 alphasatellites belonging to type 3, which share the same loop sequence with groups 1, 2, and 4, but have a distinct stem (Figure 3).

**Figure 4:**
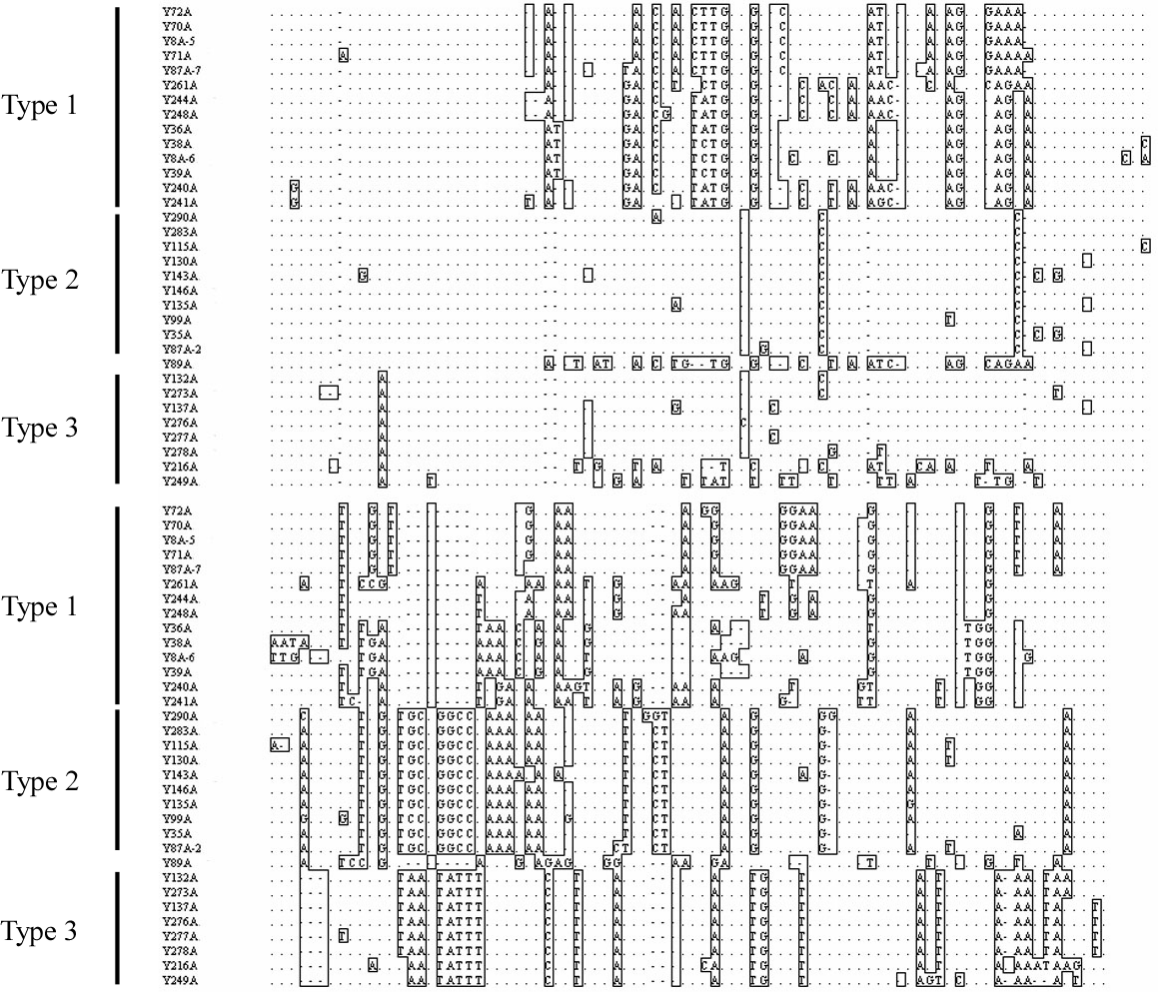
**Alignment of A-rich sequences of alphasatellite components**. Sequences that differ from each other are boxed. Gaps (-) are introduced to optimize the alignment and sequence identity is indicated with a dot (.).

A-rich regions are maintained by all alphasatellites immediately downstream of the Rep gene as reported for other alphasatellites. This A-rich region is approximately 153-169 nts long with an A-content of between 52.3-58.4%. The alignment of the sequences of the A-rich region shows that they can be divided into 3 types in accordance with the phylogenetic trees of the complete nucleotide sequences of the alphasatellites (Figure 4).

All alphasatellites encompass a single large virion-sense ORF that has the capacity to encode an approximately 36.6 kDa protein consisting of 315 amino acids, which resembles Rep of nanoviruses. Reps encoded by alphasatellites are highly conserved, with 86.3-100.0% amino acid sequence identities among the 33 alphasatellites (Table 3). Therefore, alphasatellite Rep is more conserved than complete alphasatellite sequences. Amino acid sequence comparisons of Reps also show that the 33 alphasatellites can be divided into 3 main types, which correspond to the 3 types of full-length sequence comparison (Figure 1, right).

### Mixed infection of alphasatellites

Mixed infections of geminiviruses were readily found. Some samples, including Y70-Y72, Y87, Y115, Y132, Y137, Y143, Y146, Y216, Y249, Y273, Y276-278, and Y290, were infected by 2 different viruses (Table 2). In order to determine whether each virus associated with its own alphasatellite molecule, more alphasatellites clones from these samples were sequenced. Sequence analysis revealed that mixed infections of alphasatellites occurred in samples Y87 and Y8, but not in any other samples (Table 2). Y87 was mix-infected by TbCSV and TYLCCNV, 2 alphasatellites (Y87A-2 and Y87A-7) belonging to types 1 and 2, respectively, were identified. Y8 was infected by TYLCCNV, 2 alphasatellites (Y8A-5 and Y8A-6) belonging to type 1 and sharing 85.0% nucleotide acid identity were identified. Because of the obvious divergence, we assumed that the 2 alphasatellites in Y8 were a consequence of a mixed infection by 2 distinct parental alphasatellites belonging to the same type.

## Discussion

Two single-stranded DNA components, alpha- and betasatellites, have been found to be associated with monopartite begomoviruses such as AYVV, CLCuMV, and TbCSV [4,8,11,13,23]. Betasatellites are symptom-modulating satellite molecules that depend on a helper virus for proliferation and movement. On the other hand, alphasatellites are apparently dispensable for symptomatic induction and are capable of autonomous replication [12-14]. Our results show that the 33 alphasatellites investigated are all associated with begomovirus/betasatellite complexes, which is a similar result to a report by Briddon [11]. However, only some begomovirus/betasatellite complexes were associated with alphasatellites. A better understanding of the relationship between alphasatellites and begomovirus/betasatellite complexes is achievable if future studies concentrate on the identification of alphasatellites from more symptomatic and asymptomatic crop species as well as diverse, agriculturally unimportant plant species from broader areas.

With the exception of Y89A, comparison of alphasatellites shows that they can be divided into 3 types. Type 1 alphasatellites were identified in samples infected by TYLCCNV/TYLCCNB and TYLCCNV/TYLCTHB + TYLCTHV. Since no alphasatellites were found in samples infected by TYLCTHV/TYLCTHB, it is evident that type 1 alphasatellites are associated with TYLCCNV/TYLCCNB. All type 2 alphasatellites were identified in samples infected by TbCSV/TbCSB, TbCSV/TbCSB + TbLCYNV, TbCSV/TbCSB + TYLCCNV, and TbCSV + TYLCCNV/TYLCCNB. Because no alphasatellites were found in samples infected by TbLCYNV alone, this suggests that type 2 alphasatellites are associated with TbCSV/TbCSB complexes. It is interesting that alphasatellites in sample Y146, which was mix-infected by TbCSV and TYLCCNV/TYLCCNB, were clustered in type 2 but not type 1. Sample Y146 might have been mix-infected by TYLCCNV/TYLCCNB and TbCSV/TbCSB in addition to alphasatellites associated with TbCSV/TbCSB, TbCSB then disappeared due to competition between TYLCCNB and TbCSB [24]. Most type 3 alphasatellites were found in samples mix-infected by a combination of TbCSV/AYVB and TbLCYNV or MYVYNV/MYVYNB, while 2 type 3 alphasatellites were mix-infected by TbCSV and MYVV/MYVB. Because no alphasatellites were found in samples infected by TbLCYNV, MYVYNV/MYVYNB, or MYVV/MYVB, it is apparent that type 3 alphasatellites are associated with TbCSV/AYVB. Although AYVCNV/AYVB is responsible for ageratum yellow vein disease in Hainan, China [21], AYVCNV was not found in any ageratum yellow vein disease samples in Yunnan. Instead of AYVCNV/AYVB, TbCSV/AYVB is the causal agents of ageratum yellow vein disease (Zhou et al., unpublished). It is probable that TbCSV acquires the heterogenous betasatellite, AYVB, during mixed infections, but we were unable to determine the origin of type 3 alphasatellites in this study. Sample Y89 was infected by TYLCCNV/TYLCCNB, therefore, its alphasatellite should belong to type 1. However, sequence comparison shows that Y89A shares only 69.3-79.5% nucleotide sequence identity with other alphasatellites of the 3 types. We speculate that Y89A originated from an unidentified begomovirus/betasatellite complex.

Mix-infections of begomoviruses are common; 16 of 31 isolates in this study were co-infected by 2 begomoviruses (Table 2). For most isolates, each begomovirus is associated with an alpha- and betasatellite. Two type 1 alphasatellites (Y8A-5 and Y8A-6) were identified in sample Y8, while 2 types of alphasatellites (Y87A-7 and Y87A-2) were identified in sample Y87. Our results indicate that mix-infections of alphasatellite molecules may not be unusual.

The origin of alphasatellites is undoubtedly related to nanoviruses. Presently, the function of alphasatellites is not clear, but it is evident that alphasatellites functionally interact with geminivirus/betasatellite complexes resulting in symptom alteration and a reduction in the level of viral DNA and betasatellites [12-14,25,26] Available evidence suggests that the ubiquitous association of alphasatellites with begomovirus/betasatellite complexes indicates that alphasatellites may play an important role in the occurrence, diffusion, and epidemiology of begomovirus/betasatellite complexes. More studies are required to elucidate the specific role that alphasatellites play in disease development, virus life cycle, and the evolution of begomoviruses/betasatellite complexes.

## Conclusions

Seven viruses, including TbCSV, TbLCYNV, TYLCCNV, TYLCTHV, MYVV, MYVYNV, and SLCYNV, were characterized in Yunnan Province--some of them are associated with betasatellites. Our results show that all samples from Yunnan that contained alphasatellites also had betasatellites. However, only some samples that contained betasatellites had alphasatellites. Thirty-three sequenced alphasatellites were divided into 3 types--each associated with a specific begomovirus/betasatellite complex. Type 1 was associated with TYLCCNV/TYLCCNB; type 2 was associated with TbCSV/TbCSB; and type 3 was associated with TbCSV/AYVB. Alphasatellites have 3 highly conserved structure features: a conserved hairpin structure, a single open reading frame, and an A-rich region. The alignment of the sequences of the conserved hairpin structure and the A-rich region shows that the alphasatellites can be further divided into 3 types in accordance with the phylogenetic trees of their complete nucleotide sequences. Reps encoded by the 33 alphasatellites are highly conserved and share more than 86.3% amino acid sequence identity. Alphasatellites may play an important role in the epidemiology of begomovirus/betasatellite complexes.

## Methods

### Virus sources and DNA extraction

Young seedlings were collected from naturally infected tobacco, tomato, *Ageratum conyzoides*, *Malvastrum coromandelianum*, and squash plants showing begomovirus-like infection symptoms, from locations separated by 700 km in Yunnan Province, China from 1999 to 2004. Viral DNA from the samples was extracted as previously described [20].

### PCR and sequence determination

Alphasatellite molecules were amplified by PCR with one of 2 pairs of abutting primers DNA101 (5'-CTGCAGATAATGTAGCTTACCAG-3')/DNA102 (5'-CTGCAGATCCTCCACGTGTATAG-3') or UN101 (5'-AAGCTTGCGACTATTGTATGAAAGAGG-3')/UN102 (5'-AAGCTTCGTCTGTCTTACGAGCTCGCTG-3'), which were designed from the highly conserved regions of the Rep-encoding genes of the determined alphasatellites [27]. Betasatellites were tested by PCR using abutting primers beta01 (5'-GGTACCACTACGCTACGCAGCAGCC-3') and beta02 (5'-GGTACCTACCCTCCCAGGGGTACAC-3') specific to betasatellites [28]. The PCR products were recovered, purified, and cloned using pGEM-T Easy Vector (Promega, Madison, WI, USA) as previously described [29]. Sequences were determined using an automated DNA sequencing system (Model 377; Perkin Elmer, Foster City, CA, USA).

### Sequence analysis

Sequence data were assembled and analyzed using DNAStar software version 6.0 (DNAStar Inc., Madison, WI, USA) and MEGA version 4 [30]. Sequence alignments were performed using the CLUSTAL V Multiple Sequence Alignment program in DNAStar, and phylogenetic trees were conducted using the neighbor-joining method using MEGA version 4. Other alphasatellite sequences used for comparisons were alphasatellites of *Ageratum yellow vein virus *(AYVA, AJ238493), *Cotton leaf curl Multan virus *(CLCuMA, AJ512957), *Hibiscus leaf curl virus *(HLCA, AJ512959), *Okra leaf curl virus *(OLCA, AJ512954), and *Sida yellow vein Vietnam virus *(SiYVVNA, DQ641718). Nanovirus DNA sequences used for comparisons were *Banana bunchy top virus *(BBTV AF216221), *Faba bean necrotic yellow virus *(FBNYV, X80879), *Milk vetch dwarf virus *(MVDV, AB000920), and *Subterranean clover stunt virus *(SCSV, U16736).

## Competing interests

The authors declare that they have no competing interests.

## Authors' contributions

YX, PW, PL, HG performed the experiments. YX, PW, XZ involved in data analysis and manuscript preparation. XZ provided overall direction and conducted experimental design, data analysis and wrote manuscript. All authors read and approved the final manuscript.
